# Impact of First-Line FOLFIRINOX-Induced Peripheral Neuropathy on the Efficacy of Second-Line GnP in Patients with Unresectable Advanced Pancreatic Cancer

**DOI:** 10.3390/jcm11195895

**Published:** 2022-10-06

**Authors:** Shiori Sadaka, Takuji Iwashita, Hironori Fujii, Hiroko Kato-Hayashi, Koichi Ohata, Shinya Uemura, Masahito Shimizu, Akio Suzuki

**Affiliations:** 1 Department of Pharmacy, Gifu University Hospital, 1-1 Yanagido, Gifu 501-1194, Japan; 2 First Department of Internal Medicine, Gifu University Hospital, 1-1 Yanagido, Gifu 501-1194, Japan

**Keywords:** gemcitabine, nanoparticle albumin-bound paclitaxel, adenocarcinoma, chemotherapy, peripheral neuropathy

## Abstract

Modified FOLFIRINOX (mFFX) and Gemcitabine plus nab-paclitaxel (GnP) are effective first-line chemotherapies for unresectable advanced pancreatic cancer (APC); however, both lead to peripheral neuropathy (PN). Aims: To evaluate the impact of first-line mFFX-induced PN on the efficacy of second-line GnP in patients with APC. Methods: A database containing patients with APC was retrospectively analyzed to evaluate patients who received second-line GnP after first-line mFFX failure between September 2014 and January 2021. The efficacy and safety of GnP were compared between patients with PN ≥ Grade 2 (PN group) and PN ≤ Grade 1 (non-PN group) at the start of second-line GnP. Cox proportional hazards analysis was also performed to examine the effect on overall survival (OS) and time-to-treatment failure (TTF). Results: Fifty-nine patients (PN group, 18 patients; non-PN group, 41 patients) were included. Median OS and TTF in the PN versus non-PN group were 7.7 versus 5.7 months (*p* = 0.19) and 3.8 versus 2.7 months (*p* = 0.18), respectively. Multivariate analysis showed that PN (≥Grade 2) was not a significant factor affecting either OS (hazard ratio (HR) 0.66, 95% confidence interval [CI] 0.33–1.31, *p* = 0.24) or TTF (HR 0.71, 95% CI 0.38–1.33, *p* = 0.28). No significant difference was observed in the relative dose intensity of GEM or nab-PTX, and incidence of adverse events. Conclusions: mFFX-induced PN has little impact on the efficacy and safety of second-line GnP in patients with APC. Second-line GnP could be a possible treatment option regardless of the presence of PN.

## 1. Introduction

Pancreatic cancer (PC) is the seventh leading cause of cancer-related death in the world, with a five-year survival rate of 11% [1,2]. The prognosis for PC is similarly poor in Japan, where it is the fourth leading cause of cancer-related death, with a five-year survival rate of only 8.5% [3]. While surgical resection with radical intent remains the only potentially curative treatment, only 20–30% of patients with PC are candidates for resection at diagnosis [4]. Chemotherapy is the treatment of choice for unresectable advanced pancreatic cancer (APC). In 1997, a phase III trial by Burris et al. reported that gemcitabine (GEM) was significantly superior to 5-fluorouracil (5-FU) in cancer-induced symptom palliation and 1-year survival [5]. Since this study, GEM has been considered a first-line chemotherapy regimen for APC. Recently, FOLFIRINOX (FFX; a combination of 5-FU, oxaliplatin (L-OHP), irinotecan, and leucovorin) and GEM plus nanoparticle albumin-bound paclitaxel (nab-PTX) (GnP) were shown to enable longer overall survival (OS) than GEM alone [6,7,8]. However, because FFX was reported to lead to a high incidence of grade 3–4 neutropenia (77.8%) and febrile neutropenia (22.2%) in a Japanese cohort [6], a modified FFX regimen (mFFX: reduced irinotecan dose, no bolus 5-FU) is often used for APC in Japan, since mFFX reduces the incidence of grade 3–4 neutropenia to 47.8% while maintaining efficacy [9]. Yoshida et al. reported that mFFX reduced the incidence of grade 3–4 hematologic toxicity compared to FFX, which showed efficacy comparable to FFX [10]. In APC patients with a good performance status (PS) of 0–1, as indicated by an Eastern Cooperative Oncology Group (ECOG) classification, and tolerance to intensive chemotherapy, FFX (including mFFX) or GnP is recommended as first-line chemotherapy [11]. A possible management strategy for APC in patients with better PS can comprise FFX, which is an intensive treatment that combines multiple drugs, as the primary treatment followed by GnP as the second-line treatment. A phase II study by Mita et al. evaluating the efficacy of second-line GnP therapy after failure of mFFX as first-line chemotherapy for APC showed that the OS from initiation of mFFX was 14.2 months [12], which is an improvement on an OS of 11.1 months reported in the original phase III study by Colony et al. for first-line FFX [8]. This indicates that using GnP as second-line therapy after FFX could be an effective treatment strategy.

L-OHP, a platinum-based anticancer agent used in mFFX, and nab-PTX, a taxane-based anticancer agent used in GnP, are known to cause high rates of peripheral neuropathy (PN). The incidence of PN in patients treated with mFFX and GnP is 75% (≥Grade 3: 5.6%) and 54% (≥Grade 3: 17%), respectively [6,7]. Chemotherapy-induced peripheral neuropathy (CIPN) can deteriorate patients’ quality of life (QOL) and lead to treatment discontinuation despite sufficient response. In sequential administration of mFFX followed by GnP in patients with APC, there is a concern that first-line treatment-induced PN may affect the efficacy and safety of the second-line treatment. At our institution, mFFX therapy is selected as the first-line treatment when PS, age, and other factors are considered acceptable, and GnP is used as the second-line treatment in APC patients with first-line mFFX failure. However, the effect of the severity of first-line treatment-induced PN on the efficacy and safety of the second-line treatment has not been well studied. We then conducted this study to evaluate the impact of first-line mFFX-induced PN on the efficacy of second-line GnP in patients with APC.

## 2. Materials and Methods

### 2.1. Study Design and Patient Selection

This retrospective observational study was conducted at a single center, Gifu University Hospital. We analyzed a database containing patients with APC receiving chemotherapy between September 2014 and January 2021 to identify patients who met the following criteria: (1) GnP was performed as second-line treatment after failure of FFX for APC; and (2) pancreatic cancer was pathologically proven to be ductal adenocarcinoma. No exclusion criteria were set for this study. The efficacy and safety of GnP were compared between the two groups at the start of GnP in patients with PN ≥ Grade 2 (PN group) and PN ≤ Grade 1 (non-PN group). Tumor response was evaluated using computed tomography (CT) scans as complete response (CR), partial response (PR), stable disease (SD), or progressive disease (PD). Treatment response was assessed according to the Response Evaluation Criteria in Solid Tumors (RECIST) version 1.1 [13]. The overall response rate (ORR) was defined as CR plus PR, and the disease control rate (DCR) as CR + PR + SD.

### 2.2. CIPN Evaluation

CIPN was assessed using the National Cancer Institute Common Terminology Criteria for Adverse Events (CTCAE) version 5.0 [14]. Grade 1 was defined as no symptoms (decreased deep tendon reflexes or abnormal perception), Grade 2 as moderately symptomatic (limitation of activities of daily living except for personal activities), and Grade 3 as severely symptomatic (limitation of activities of daily living including personal activities). Limitations to activities of daily living in Grade 2 CIPN are not directly related to food, clothing, shelter, or labor, but include texting on cell phones, carrying heavy objects, flipping through books and magazines, and exercising or playing musical instruments. Meanwhile, limitations to activities of daily living in Grade 3 CIPN can be directly related to food, clothing, shelter, and labor, such as opening and closing buttons, eating with chopsticks, climbing stairs, putting on shoes, and grasping objects. The treating physician and pharmacist evaluated the degree of PN before each administration of chemotherapy including at the timing of the initiation of GnP and documented it in the medical record.

### 2.3. Statistical Analyses

The primary endpoint was OS. Secondary endpoints were a time-to-treatment failure (TTF), response rate (RR), relative dose intensity (RDI), adverse events (AEs), and change in the severity of PN. OS was calculated from the start date of second-line GnP to the date of death. TTF was calculated from the start date of second-line GnP to the date of GnP discontinuation. Patients without the event of death or treatment discontinuation were censored at the time of the last follow-up. During the treatment period, patients were assessed for their general condition and AEs by physical and blood examinations. AEs were graded based on the CTCAE version 5.0 [14]. The most severe grades of AEs observed during GnP were recorded.

Continuous variables are expressed as median (range) and were compared using the Mann–Whitney U test. Fisher’s exact test and Pearson’s chi-squared test were used to compare categorical variables. OS and TTF were estimated using the Kaplan–Meier method and compared using the log-rank test. For each test, *p* values lower than 0.05 were considered statistically significant. The Cox proportional hazards model was used to examine factors associated with OS and TTF. Possible factors included the presence or absence of PN, age, sex, metastasis, CA19-9 (carbohydrate antigen 19-9), and cumulative dose of L-OHP (mg/m^2^). All statistical analyses were performed using EZR (Saitama Medical Center, Jichi Medical University, Saitama, Japan) and GraphPad Prism version 9.3.1 (GraphPad Software, San Diego, CA, USA).

### 2.4. Ethics Statement

This study was conducted in accordance with the guidelines for human studies adopted by the ethics committee of Gifu University Graduate School of Medicine and notified by the Japanese government (institutional review board approval no. 2021-B173). In view of the retrospective nature of the study, subject informed consent was not required. All procedures performed in studies involving human participants were in accordance with the ethical standards of the institutional and/or national research committee and with the 1964 Helsinki declaration and its later amendments or comparable ethical standards.

## 3. Results

### 3.1. Patient Characteristics

**Figure 1:**
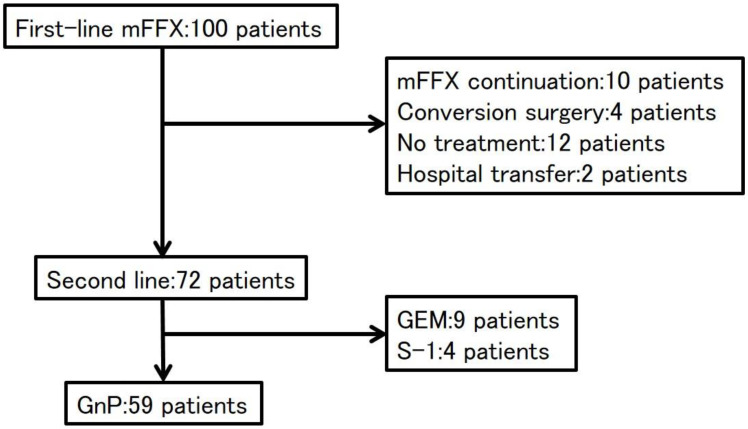
Consort diagram. mFFX: modified FOLFIRINOX, GEM: gemcitabine, GnP: gemcitabine plus nab-paclitaxel.

During the study period, 100 patients with APC received mFFX as first-line treatment. Ten patients continued with mFFX to the end of the study period and four patients underwent conversion surgery because of a good response to mFFX. After treatment failure with first-line mFFX, 72 patients underwent second-line treatment, 12 patients did not receive further treatment, and 2 patients were transferred to other hospitals. Second-line regimens included GnP in 59 patients, GEM in 9 patients, and S-1 in 4 patients. The reasons for no GnP regimen as second-line were the deteriorated PS in 12 patients and CIPN of grade 2 in 1 patient. The 59 patients who underwent GnP as second-line treatment were included in our analysis (Figure 1). Of the 59 patients, 13 patients did not have PN while the severity of PN was Grade 1 in 28 patients and Grade 2 in 18 patients. Thus, the PN group included 18 patients and the non-PN group had 41 patients.

The baseline characteristics of the patients are shown in Table 1. There were no significant differences between the groups except in two factors: the median cumulative dose of L-OHP was significantly higher in the PN group than in the non-PN group (857.5 mg/m^2^ versus 617.4 mg/m^2^, *p =* 0.03) and the proportion of patients taking pregabalin/mirogabalin-containing treatment was significantly higher in the PN group compared to the non-PN group (50% versus 7.3%, *p* < 0.001).

### 3.2. Efficacy of Chemotherapy

**Figure 2:**
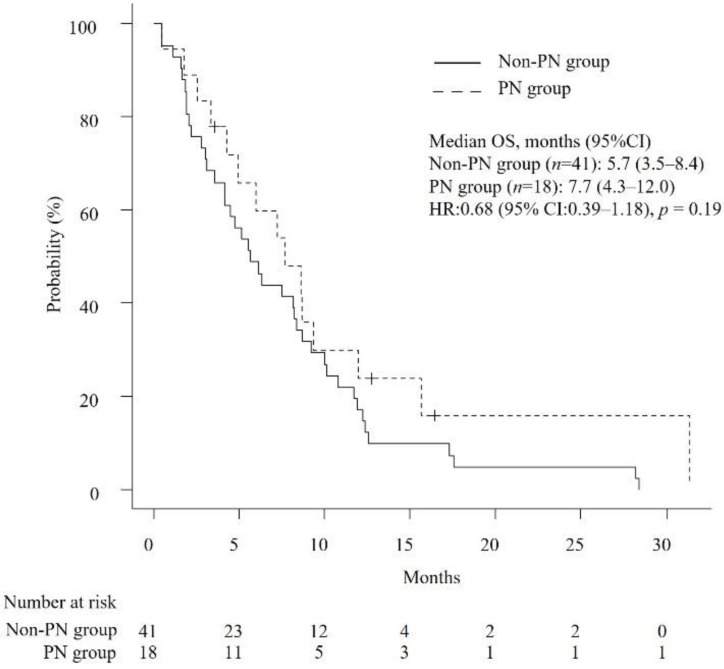
Kaplan–Meier curves comparing the overall survival of patients with unresectable APC who received second-line GnP. PN: peripheral neuropathy, OS: overall survival, HR: hazard ratio.

**Figure 3:**
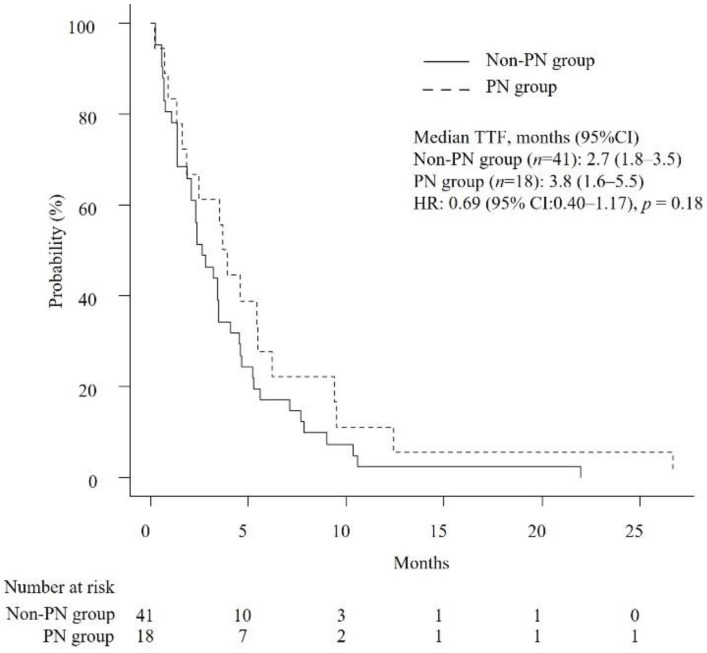
Kaplan–Meier curves comparing the time-to-treatment failure of patients with unresectable APC who received second-line GnP. PN: peripheral neuropathy, TTF: time-to-treatment failure, HR: hazard ratio.

The median OS and TTF were 6.3 months (95% confidence interval [CI]: 4.7–8.6) and 3.2 months (95% CI: 2.1–3.9), respectively. As shown in Table 2, the median number of chemotherapy cycles received by patients in the PN group and the non-PN group was 4.5 and 3, respectively (*p* = 0.39). The median RDIs of GEM and nab-PTX were not significantly different between the PN group and non-PN group (GEM, 0.75 versus 0.71, *p* = 0.88; nab-PTX, 0.63 versus 0.7, *p* = 0.53). Likewise, median OS and TTF were not significantly different (OS, 7.7 months versus 5.7 months, *p* = 0.19; TTF, 3.8 months versus 2.7 months, *p* = 0.18) (Figure 2 and Figure 3). Cox proportional hazards analysis showed that none of the studied factors had a significant effect on OS and TTF, including the occurrence of PN at the start of second-line GnP (OS, hazard ratio (HR) of 0.66 with 95% CI of 0.33–1.31, *p* = 0.24; TTF, HR of 0.71 with 95% CI of 0.38–1.33, *p* = 0.28) (Table 3). The ORR was 0% and 4.87% (*p* = 1.0) and the DCR was 38.9% and 36.6% in the PN group and non-PN group (*p* = 1.0), respectively.

### 3.3. Safety Profile

The incidence of AEs ≥ Grade 3 was not significantly different between the PN group and non-PN group (72.2% versus 87.8%, *p* = 0.27). As shown in Table 4, non-hematologic toxicity, a ≥Grade 3 AE of PN, tended to be higher in the PN group than in the non-PN group (5.6% versus 0%, *p* = 0.31). There were no significant differences in other Grade 3 or higher AEs between the two groups. Reasons for discontinuation of GnP (PN versus non-PN group) were PD in 16 patients (88.9%) versus 39 patients (95.1%), and AEs in 2 patients (11.1%: interstitial pneumonia in 2) versus 2 patients (4.87%: interstitial pneumonia in 1 and eye disorder in 1). The change in the severity of PN from the start to the end of GnP therapy is shown in Table 5. Progression of PN from the start to the end of GnP was observed in one patient (5.6%: Grade 2 to Grade 3) in the PN group and in eight patients (19.5%: Grade 0/1 to Grade 2) in the non-PN group. No patients experienced an interruption to GnP treatment due to deterioration of PN.

## 4. Discussion

This study evaluated the impact of first-line mFFX-induced PN on the efficacy of second-line GnP in patients with APC. Median OS and TTF were not significantly different between the PN and non-PN groups. This finding was confirmed using Cox proportional hazards analysis, which showed that the severity of PN at the start of second-line GnP was not a significant factor affecting OS or TTF. Although ≥Grade 3 AEs occurred in ≥70% of patients in both groups during the clinical course of GnP treatment, no discontinuation of GnP due to PN was observed.

To date, no phase III trials have examined the effectiveness of chemotherapy regimens administered after the failure of mFFX or GnP as first-line treatment in pancreatic cancer patients. Nevertheless, the NCCN guidelines recommend GEM-based therapy for patients with good PS after the failure of fluoropyrimidine-based therapy [11]. Several studies that have evaluated the efficacy and safety of second-line GnP in patients with APC after the failure of first-line FFX have demonstrated a median OS of 5.2–9.9 months, median PFS of 2.8–5.1 months, RR of 9.2–17.9%, and DCR of 46–87.5% (Table 6) [12,15,16,17,18]. In this study, median OS (6.3 months, 95% CI: 4.7–8.6) and TTF (3.2 months, 95% CI: 2.1–3.9) were consistent with values reported by Zang et al. [16] and Mita et al. [12]. However, while the incidence rate of AEs ≥ Grade 3 was around 40–70% in previous reports [12,15,16,17,18], it was as high as 84.7% in our study. Most ≥Grade 3 hematologic toxicities were manageable by reducing the doses of GEM or nab-PTX while continuing GnP treatment. The main reason for reducing the dose of nab-PTX was neutropenia (≥Grade 3), which had an incidence of 55.6% in the PN group and 58.5% in the non-PN group. It is possible that patients with APC who receive GnP as second-line therapy may experience bone marrow exhaustion due to myelosuppression caused by first-line treatment with mFFX. Therefore, a more significant impact of GnP on RDI may not be PN but rather bone marrow suppression. These results suggest that GnP could be a second-line chemotherapy option for patients who have experienced treatment failure with mFFX.

However, despite the potential of GnP as a second-line treatment option, there is a possibility that first-line mFFX-induced PN could affect the efficacy and safety of second-line GnP in patients with APC. In this study, we showed that the severity of PN at the initiation of second-line GnP did not affect the drug’s efficacy or safety after the failure of first-line mFFX. Progression of PN during GnP treatment was observed in only one patient (5.6%: Grade 2 to Grade 3) in the PN group, although it tended to occur more frequently in the non-PN group (19.5%: Grade 0/1 to Grade 2). However, no treatment discontinuation occurred because of PN-related reasons. Yamaguchi et al. studied the change in PN in patients with advanced gastric cancer who received first-line L-OHP-based regimens and second-line PTX treatment, which is similar to the mFFX followed by the GnP regimen examined in this study. The severity of PN at the end of first-line treatment (≥Grade 2 versus ≤Grade 1) did not significantly affect the incidence rate of PN ≥ Grade 2 during second-line PTX [19]. These results suggest that L-OHP-induced PN may not affect the incidence of PN associated with taxane-based anticancer therapy. The severity of L-OHP and PTX-induced PN is known to depend on each drug’s cumulative dosage [20,21]. Further, the mechanism underlying PN differs between platinum-based and taxane-based anticancer agents. In general, platinum-based anticancer agents such as L-OHP directly damage neurons, resulting in secondary damage in the form of axonal degeneration and demyelination. In contrast, taxanes such as PTX damage axons, while neuronal cell bodies remain relatively preserved [22]. Considering the high feasibility of sequential administration of L-OHP followed by PTX regardless of the degree of PN, and the mechanistic differences by which these drugs cause PN, the presence of L-OHP-induced PN may not be a criterion for subsequent treatment with PTX-related regimens. Further evaluation is required to confirm this theory.

In terms of the management of PN, a previous study reported the efficacy of duloxetine as supportive therapy for CIPN, especially with platinum-based anticancer agents [22]. Pregabalin reportedly improves CIPN symptoms in patients receiving L-OHP-containing regimens [23]. In the present study, the proportion of patients taking pregabalin or mirogabalin at the start of GnP was significantly higher in the PN group (nine patients (50%)) than that in the non-PN group (three patients (7.3%)) (*p* < 0.001). Considering that 50% of patients in the PN group received pregabalin or mirogabalin, it is possible that pregabalin or mirogabalin may have reduced the symptoms of PN or prevented potential progression caused by taxanes. Based on these results, early supportive therapy with duloxetine, pregabalin, and mirogabalin for L-OHP-induced PN might be important for maintaining the efficacy and safety of second-line GnP after the failure of mFFX in patients with APC.

This study has several limitations. First, this was a retrospective study that examined a small cohort at a single center. This study design may be associated with bias in patient selection and limit external validity. Second, since this study did not include patients with PN ≥ Grade 3, the effect of GnP on more severe PN could not be evaluated. Third, there may have been potential discrepancies between assessments by medical staff and actual PN symptoms, since PN is reportedly one of the most subjective AEs and is often prone to underestimation by medical staff [24]. Use of the Numerical Rating Scale (NRS) and Visual Analogue Scale (VAS) in addition to CTCAE in the future could improve the accuracy and reduce discrepancies when evaluating the severity of PN.

In conclusion, the severity of PN induced by first-line mFFX likely has little impact on the efficacy and safety of second-line GnP in patients with APC. Therefore, second-line GnP administered after mFFX may be an effective treatment option regardless of the presence of PN, although further large-scale studies are required to confirm our findings.

## Figures and Tables

**Table 1:** Demographics and baseline characteristics of patients in the PN and non-PN group at the start of GnP.

	PN Group (*n* = 18)	Non-PN Group (*n* = 41)	*p*-Value
Sex			
Male	12 (66.7%)	18 (43.9%)	0.184 ^a^
Female	6 (33.3%)	23 (56.1%)	
Age, median (min–max)	66 (42–75)	64 (39–77)	0.650 ^b^
Height, cm	165 (159–168)	158 (154.1–163)	0.049 ^b^
Weight, kg	55 (48–68)	50.0 (47.5–58.9)	0.162 ^b^
ALB, g/dL	3.9 (3.5–3.9)	3.6 (3.2–3.9)	0.248 ^b^
NLR	2.74 (2.01–3.21)	2.5 (1.9–4.3)	0.967 ^b^
mGPS	11/5/2	14/17/10	0.145 ^a^
CA19-9, U/mL	2452 (493.4–5609.4)	1741.5 (428.8–3786.3)	0.775 ^b^
Metastasis	17 (94.4%)	36 (87.8%)	0.656 ^a^
Diabetes mellitus	9 (50%)	14 (34.1%)	0.390 ^a^
Cumulative dose of oxaliplatin, mg/m^2^	857.5 (778.1–1091.1)	617.4 (327.8–1018.9)	0.030 ^b^
Pregabalin or mirogabalin	9 (50%)	3 (7.3%)	0.001 ^c^
Duloxetine	1 (5.6%)	0	0.305 ^c^
Nab-PTX initial dose			
Full dose (125 mg/m^2^)	4 (22.2%)	16 (39%)	0.323 ^a^
Level 1 (100 mg/m^2^)	13 (72.2%)	21(51.2%)	
Level 2 (75 mg/m^2^)	1 (5.6%)	4 (9.8%)	

PN: peripheral neuropathy, GnP: gemcitabine plus nab-paclitaxel, ALB: albumin, NLR: Neutrophil-to-Lymphocyte Ratio, mGPS: modified Glasgow Prognostic Score, CA19-9: carbohydrate antigen 19-9, Nab-PTX: nanoparticle albumin-bound paclitaxel. ^a^ *Chi-squared test*, ^b^ *Mann–Whitney U-test*, ^c^ *Fisher’s exact test*. Data indicate median with 25–75th percentiles or numbers.

**Table 2:** Comparison of tumor response, relative dose intensity, and the number of cycles.

	PN Group (*n* = 18)	Non-PN Group (*n* = 41)	*p*-Value
Overall survival, months	7.7	5.7	0.198 ^a^
Time-to-treatment failure, months	3.8	2.7	0.180 ^a^
Response rate (CR + PR), *n* (%)	0	2 (4.87%)	1.00 ^b^
Complete response, *n* (%)	0	0	1.00 ^b^
Partial response, *n* (%)	0	2 (4.87%)	1.00 ^b^
Stable disease, *n* (%)	7 (38.9%)	13 (31.7%)	0.81 ^c^
Progressive disease, *n* (%)	11 (61.1%)	26 (63.4%)	0.78 ^c^
DCR (CR + PR + SD), *n* (%)	7 (38.9%)	15 (36.6%)	1.00 ^c^
Relative dose intensity			
GEM	0.75	0.71	0.88 ^a^
Nab-PTX	0.63	0.7	0.53 ^a^
Number of cycles, median (min–max)	4.5 (1–29)	3 (1–25)	0.39 ^a^

PN: peripheral neuropathy, CR: complete response, PR: partial response, DCR: disease control rate, SD: stable disease, GEM: gemcitabine, Nab-PTX: nanoparticle albumin-bound paclitaxel. ^a^ *Mann–Whitney U-test*, ^b^ *Fisher’s exact test*, ^c^ *Chi-squared test*.

**Table 3:** Cox proportional hazards analyses of OS and TTF in unresectable APC patients receiving second-line GnP.

Factor	OS			TTF		
	HR	(95% CI)	*p*-Value	HR	(95% CI)	*p*-Value
PN (≥Grade 2)	0.66	(0.33–1.31)	0.24	0.71	(0.38–1.33)	0.28
Age (≥64)	0.66	(0.36–1.21)	0.18	0.89	(0.50–1.59)	0.71
Sex (Female)	1.27	(0.69–2.35)	0.48	1.29	(0.71–2.37)	0.40
Metastasis (Yes)	1.16	(0.44–3.07)	0.78	1.69	(0.62–4.36)	0.32
CA19-9 (≥1741.5 U/mL)	0.87	(0.50–1.52)	0.63	0.98	(0.57–1.66)	0.93
Cumulative dose of oxaliplatin (≥800 mg/m^2^)	1.14	(0.61–2.05)	0.73	0.96	(0.53–1.74)	0.90

OS: overall survival, TTF: time-to-treatment failure, APC: advanced pancreatic cancer, HR: hazard ratio, CI: confidence interval, PN: peripheral neuropathy, CA19-9: carbohydrate antigen 19-9.

**Table 4:** Comparison of the incidence of adverse events (≥Grade 3) between the PN and non-PN groups.

Adverse Event (≥Grade 3)		PN Group (*n* = 18)	Non-PN Group (*n* = 41)	*p*-Value
Hematological, *n* (%)	Leukopenia	8 (44.4)	23 (56.1)	0.59 ^a^
	Neutropenia	10 (55.6)	24 (58.5)	1.00 ^a^
	Anemia	4 (22)	20 (48.8)	0.08 ^b^
	Thrombocytopenia	3 (16.7)	11 (26.8)	0.52 ^b^
	Febrile neutropenia	0	5 (12.2)	0.31 ^b^
Non-hematological, *n* (%)	Nausea	0	1 (2.4)	1.00 ^b^
	Vomiting	0	0	1.00 ^b^
	Dysgeusia	0	0	1.00 ^b^
	Stomatitis	0	0	1.00 ^b^
	Diarrhea	0	2 (4.9)	1.00 ^b^
	Peripheral neuropathy	1 (5.6)	0	0.31 ^b^
	Fatigue	0	0	1.00 ^b^

PN: peripheral neuropathy. ^a^ *Chi-squared test*, ^b^ *Fisher’s exact test*.

**Table 5:** Progression of PN during treatment with second-line GnP.

Change in Severity of PN (GnP Start → End)	PN Group (*n* = 18)	Non-PN Group (*n* = 41)
G0 → G0	-	4 (9.8%)
G0 → G1	-	7 (17.1%)
G0 → G2	-	2 (4.9%)
G0 → G3	-	0
G1 → G0	-	1 (2.4%)
G1 → G1	-	21 (51.2%)
G1 → G2	-	6 (14.6%)
G1 → G3	-	0
G2 → G0	0	-
G2 → G1	2 (11.1%)	-
G2 → G2	15 (83.3%)	-
G2 → G3	1 (5.6%)	-

PN: peripheral neuropathy, GnP: gemcitabine plus nab-paclitaxel.

**Table 6:** Available studies on second-line GnP after the failure of first-line FFX.

Author, Year	Design	Number of Patients	RR	DCR	Median OS (Months)	Median PFS (Months)	RDI (Nab-PTX)	AE (≥Grade 3)	PN (≥Grade 3)	Discontinuation of PN
Portal et al., 2015 [15]	Prospective	57	17.5%	58%	8.8	5.1	0.59	40%	12.5%	7%
Zhang et al., 2015 [16]	Retrospective	28	17.9%	46%	5.2	2.8 (TTF)	0.58	-	-	3.6%
Chae et al., 2019 [17]	Retrospective	102	9.2%	73.6%	9.8	4.6	-	60.2%	8.2%	-
Huh et al., 2021 [18]	Phase Ⅱ	40	15%	87.5%	9.9	5.8	-	62.5%	10%	5%
Mita et al., 2019 [12]	Phase Ⅱ	30	13.3%	46.7%	7.6	3.8	0.67	70%	13.3%	0

GnP: gemcitabine plus nab-paclitaxel, FFX: FOLFIRINOX, RR: response rate, DCR: disease control rate, OS: overall survival, PFS: progression-free survival, RDI: relative dose intensity, AE: adverse event, PN: peripheral neuropathy, TTF: time-to-treatment failure.

## Data Availability

Data is available by contacting the correspondence author.

## Abbreviations

APC, advanced pancreatic cancer; CI, confidence interval; DCR, disease control rate; FFX, FOLFIRINOX; mFFX, modified FOLFIRINOX; 5-FU, 5-fluorouracil; GnP, gemcitabine plus nab-paclitaxel; HR, hazard ratios; CA19-9, carbohydrate antigen 19-9; NRS, numerical rating scale; PN, peripheral neuropathy; ORR, overall response rate; OS, overall survival; L-OHP, oxaliplatin; QOL, quality of life; RDI, relative dose intensity; RR, response rate; TTF, time-to-treatment failure; VAS, visual analogue scale.
